# Active immunization with myelin-derived altered peptide ligand reduces mechanical pain hypersensitivity following peripheral nerve injury

**DOI:** 10.1186/s12974-015-0253-4

**Published:** 2015-02-13

**Authors:** Chamini J Perera, Samuel S Duffy, Justin G Lees, Cristina F Kim, Barbara Cameron, Vasso Apostolopoulos, Gila Moalem-Taylor

**Affiliations:** School of Medical Sciences, University of New South Wales, UNSW Medicine, Sydney, NSW 2052 Australia; Centre for Infection and Inflammation Research, School of Medical Sciences, University of New South Wales, Sydney, NSW 2052 Australia; College of Health and Biomedicine, Centre for Chronic Disease Prevention and Management, Victoria University, Melbourne, VIC Australia

**Keywords:** Neuropathic pain, Myelin basic protein, Altered peptide ligand, Mechanical allodynia, Pro-inflammatory cytokines, Anti-inflammatory cytokines, M1 and M2 macrophages

## Abstract

**Background:**

T cells have been implicated in neuropathic pain that is caused by peripheral nerve injury. Immunogenic myelin basic protein (MBP) peptides have been shown to initiate mechanical allodynia in a T cell-dependent manner. Antagonistic altered peptide ligands (APLs) are peptides with substitutions in amino acid residues at T cell receptor contact sites and can inhibit T cell function and modulate inflammatory responses. In the present study, we studied the effects of immunization with MBP-derived APL on pain behavior and neuroinflammation in an animal model of peripheral nerve injury.

**Methods:**

Lewis rats were immunized subcutaneously at the base of the tail with either a weakly encephalitogenic peptide of MBP (cyclo-MBP_87-99_) or APL (cyclo-(87-99)[A^91^,A^96^]MBP_87-99_) in complete Freund’s adjuvant (CFA) or CFA only (control), following chronic constriction injury (CCI) of the left sciatic nerve. Pain hypersensitivity was tested by measurements of paw withdrawal threshold to mechanical stimuli, regulatory T cells in spleen and lymph nodes were analyzed by flow cytometry, and immune cell infiltration into the nervous system was assessed by immunohistochemistry (days 10 and 30 post-CCI). Cytokines were measured in serum and nervous tissue of nerve-injured rats (day 10 post-CCI).

**Results:**

Rats immunized with the APL cyclo-(87-99)[A^91^,A^96^]MBP_87-99_ had significantly reduced mechanical pain hypersensitivity in the ipsilateral hindpaw compared to cyclo-MBP_87-99_-treated and control rats. This was associated with significantly decreased infiltration of T cells and ED1+ macrophages in the injured nerve of APL-treated animals. The percentage of anti-inflammatory (M2) macrophages was significantly upregulated in the APL-treated rats on day 30 post-CCI. Compared to the control rats, microglial activation in the ipsilateral lumbar spinal cord was significantly increased in the MBP-treated rats, but was not altered in the rats immunized with the MBP-derived APL. In addition, immunization with the APL significantly increased splenic regulatory T cells. Several cytokines were significantly altered after CCI, but no significant difference was observed between the APL-treated and control rats.

**Conclusions:**

These results suggest that immune deviation by active immunization with a non-encephalitogenic MBP-derived APL mediates an analgesic effect in animals with peripheral nerve injury. Thus, T cell immunomodulation warrants further investigation as a possible therapeutic strategy for the treatment of peripheral neuropathic pain.

## Background

Peripheral nerve injury often results in neuropathic pain, a chronic debilitating condition that adversely affects the quality of life of sufferers. It is characterized by spontaneous pain and stimulus-evoked pain, including allodynia (pain due to a stimulus which does not normally provoke pain) and hyperalgesia (an increased response to a stimulus which is normally painful) [1]. Neuropathic pain is known to be associated with significant pathological changes in the nervous system. A growing body of evidence suggests that neuroinflammation plays a vital role in the pathogenesis of such pain; immune cells (such as macrophages and lymphocytes), glial cells in the periphery and microglia and astrocytes in the spinal cord, as well as pro-inflammatory cytokines secreted by these cells, have all been implicated [2-4]. In particular, recent studies have demonstrated that T cells contribute to neuropathic pain following nerve injury [5,6].

T cells are one of the major cellular components of the adaptive immune response. They are a heterogeneous group, divided into helper T (Th) cells, cytotoxic T cells, and regulatory T (Treg) cells, with several subpopulations of each type. T cells are almost absent in the intact nervous system tissue. Following peripheral nerve injury, T cells infiltrate the injured nerve [6,7], the dorsal root ganglia (DRGs) [8,9], and the spinal cord [5,8,10]. Athymic nude rats that lack mature T cells [6], nude and CD4-knockout mice [10], and T cell-deficient Rag1-null mice [5] display reduced neuropathic pain behaviors. The precise role of specific T cell subsets in neuropathic pain remains unclear, but at least in part depends on their distinctive cytokine profile. The phenotype of spinal cord-infiltrating CD4+ T cells has been characterized as a pro-inflammatory Th1 subtype in a murine model of neuropathic pain [11]. Th1 cells producing pro-inflammatory cytokines such as interferon-γ (IFN-γ) and Th17 cells producing the pro-inflammatory cytokine IL-17 have been shown to contribute to neuropathic pain hypersensitivity [5,6,12]. In contrast, Th2 cells producing anti-inflammatory cytokines (e.g., interleukin; IL-10) have been shown to attenuate pain hypersensitivity [6]. Furthermore, expansion of immunosuppressive Treg cells by treatment with CD28 superagonist decreased, whereas suppression of Treg cells by anti-CD25 antibody treatment increased mechanical allodynia following peripheral nerve injury [13]. Thus, modulation of the T cell immune response in the nervous system may be useful in the management of chronic neuropathic pain.

Altered peptide ligands (APLs) (also known as mutant or antagonist peptides) are analogs derived from the original antigenic peptide that commonly carry a single or more amino acid substitutions at T cell receptor (TCR) contact residues. Engagement of the TCR by such APLs usually impairs normal T cell function [14]. Thus, some APLs are able to specifically antagonize and inhibit T cell activation induced by the wild-type antigenic peptide [15]. APLs have been shown to modulate the immune response both *in vitro* and *in vivo* by different cellular mechanisms including mediating partial agonism, antagonism and T cell anergy [16], shifting immune responses from Th1 to Th2 [17], down-regulation of pro-inflammatory cytokines (e.g., tumor necrosis factor; TNF and IFN-γ) and up-regulation of anti-inflammatory cytokines (e.g., IL-4, IL-10, and tumor growth factor-β) [18,19], and bystander suppression by induction of protective Treg cells [20]. Indeed, APLs have been used as immunotherapeutics in autoimmune diseases, infectious diseases, and cancer [15]. For example, APLs derived from proteins of the myelin sheath are used in experimental autoimmune encephalomyelitis (EAE), a chronic demyelinating autoimmune disease of the central nervous system which serves as an animal model of multiple sclerosis (MS). Immunization with immunodominant epitopes of myelin basic protein (MBP) or myelin proteolipid protein (PLP) induces EAE. In the last two decades, many studies in rodents have demonstrated that several MBP- or PLP-derived APLs confer protection from the development of EAE and even reverse established paralytic disease [16,18,21]. These APLs were shown to induce T cells that are cross-reactive with the native myelin peptide, but modify the immune response and prevent autoimmune encephalomyelitis.

In this study, we chose to use a MBP-derived APL. MBP is a major constituent of the myelin sheath of oligodendrocytes and Schwann cells in the nervous system and is considered to be a key auto-antigen in inducing T cell activation in MS and EAE [22,23]. We have previously demonstrated that active immunization with MBP-derived APL in an animal model of EAE not only mitigated the disease course but also improved symptoms of mechanical pain hypersensitivity in rats [24]. In addition, recent studies have shown that immunization with MBP-derived APL significantly protected from neuronal loss and promoted recovery after incomplete spinal cord injury [25] and ameliorated depressive behavior in an animal model of depression [26] by provoking a protective immune response. Furthermore, exposure of immunodominant MBP peptide epitopes by proteolysis of myelin via matrix metalloproteinases in the injured nerve induced mechanical allodynia in both a T cell-dependent and independent manner [27,28]. Considering that MBP peptides are able to initiate mechanical hypersensitivity [28] and that MBP-derived APLs can modulate the MBP-specific T cell immune response, we studied the effects of immunization with MBP-derived APL on pain hypersensitivity, immune cell reactivity, prevalence of regulatory T cells, and cytokine expression profile in neuropathic animals following chronic constriction injury (CCI) of the sciatic nerve, a widely accepted animal model of peripheral neuropathy.

## Methods

### Animals

Male Lewis rats 8 to 10 weeks old were used in all experiments (Animal Resource Centre, Perth, WA, Australia). The rats were group-housed with water and food *ad libitum* and maintained on a 12:12 h light/dark cycle. The facility was kept at a constant room temperature and humidity level, and the animals were monitored daily throughout the experiments. All experiments in animals were approved by the Animal Care and Ethics Committee of the University of New South Wales, Sydney, Australia and performed in compliance with guidelines issued by the International Association for the Study of Pain.

### Chronic constriction injury of the sciatic nerve

The rats were anesthetized with 5% isofluorane (Delvet Pty Ltd., Seven Hills, NSW, Australia) and were maintained with 2% to 3% of isoflurane in oxygen. The surgical site was aseptically prepared, and CCI was performed on the left sciatic nerve (LSN) according to the protocol of Bennette and Xie [29]. Briefly, the thigh skin was incised, the connective tissue between the gluteus superficialis and the biceps femoris muscles was cut, and the left sciatic nerve was exposed and freed. Four loose ligatures were tied around the nerve using chromic gut sutures (4-0 Ethicon, Somerville, NJ, USA), proximal to the trifurcation without arresting the epineural blood flow. In the sham-operated rats, the nerve was exposed but not ligated. The muscle layers and subcutaneous tissue was sutured with 5-0 silk (Mersilk, Ethicon, Somerville, NJ, USA) and the incision was closed with staples (9 mm, Autoclips, BD Diagnostic, North Ryde, NSW, Australia).

### Immunization and animal monitoring

Following CCI or sham operation, the rats were immunized subcutaneously at the base of the tail (on day 0; D0) with either 200 μl of complete Freund’s adjuvant (CFA; as control; *n* = 6) or 200 μl of APL (250 μg cyclo-(87-99)[A^91^_,_A^96^]MBP_87-99_ in CFA; *n* = 6) or 200 μl of MBP (250 μg Cyclo-MBP_87-99_ in CFA; *n* = 6). CFA was prepared by mixing 1 mg/mL *Mycobacterium tuberculosis* (strain 37A, Difco Laboratories, Detroit, MI, USA) in incomplete Freund’s adjuvant (Difco Laboratories, Detroit, MI, USA). Since intraplantar administration of CFA by itself can cause inflammatory pain at the injected paw and spinal glial activation [30], we immunized animals at the base of the tail. We cannot exclude the possibility of potential effects of the CFA vehicle on pain sensitivity and neuroinflammation in our immunized rats; however, this effect would be evident in all groups. MBP_87-99_ was used as the native peptide control based on its weakly encephalitogenic property [31] and its potential ability to initiate neuroinflammation and pain on its own [28]. Previous studies using site-directed mutagenesis to compare different mutant peptides have shown that the peptide MBP_87-99_ with double Ala mutations at positions 91,96-[A^91^,A^96^]MBP_87-99_ alters immune responses leading to decreased EAE severity [32]. Our study was designed to assess the effects of this mutant peptide on pain sensitivity in the nerve-injured rats. In addition, cyclic peptides have been shown to be more stable than their linear counterparts *in vivo* [23]. Thus, for increased stability, the peptides were cyclized from head to tail. Both cyclo-MBP_87-99_ and cyclo-(87-99)[A^91^_,_A^96^]MBP_87-99_ were custom synthesized (Mimotopes Pty Ltd., Clayton, VIC, Australia). Following CCI and immunization, the rats were monitored daily for clinical signs of EAE for 4 weeks using a scale ranging from ‘0’ being clinically normal to ‘5’ being paralysis of all limbs (quadriplegia).

### Pain hypersensitivity testing

Animals were acclimatized to the pain behavioral testing apparatus for 1 h prior to the initial testing in a quiet and well-controlled environment. Thereafter, tests were carried out twice a week, after habituating the animals in the apparatus for at least 30 min. Baseline data prior to surgery and up to 4 weeks post-surgery were collected for mechanical withdrawal thresholds in hindpaws. The rats were placed in a perspex test cage on an elevated mesh, and the mid-plantar surface of the hindpaws was stimulated with a dynamic von Frey anesthesiometer (Ugo Basile, Comero, Italy). This device lifts a filament to mechanically stimulate the hindpaw with an increasing force by applying pressure to the paw, and upon withdrawal reaction, the system automatically records and displays the maximum force applied (in grams). The left and right hindpaws were tested three times in each testing session with a 3- to 5-min interval, and then the average per each paw was calculated.

### Immunohistochemistry

The rats were killed and tissues were removed for immunohistochemistry at day 10 and day 30 post-CCI. Each rat was injected with sodium pentobarbital and perfused with heparinised saline (0.9% NaCl) followed by 4% paraformaldehyde (PFA). Left and right sciatic nerves, left and right L4 and L5 DRG, and lumbar spinal cords (segment L4-L6) were dissected and post-fixed in 4% PFA overnight at 4°C and then transferred to 30% sucrose + 0.1% sodium azide solution and stored at 4°C until sectioning. Sciatic nerves and DRGs were cryosectioned longitudinally (10 μm thick), while spinal cords were sectioned coronally (20 μm thick). Sections were collected directly onto gelatine coated glass slides, air dried, and stored at −20°C until use.

For staining, slides were first incubated with 100% ethanol for 10 min at room temperature followed by two washes with dH_2_O for 5 min each. After washing with phosphate-buffered saline (PBS) for 3 min, sections were blocked for 30 min at room temperature with PBS containing 0.05% Tween 20 and 5% normal donkey serum (Jackson Immune Research, Westgrove, PA, USA). For TCRαβ staining, sections were incubated with acetone for 3 min and washed with PBS three times prior to adding the blocking solution. Sciatic nerve and DRG sections were incubated with the following primary antibodies diluted in PBS containing 5% bovine serum albumin (Sigma-Aldrich, Sydney, Australia) and 0.05% Tween-20 for 1 h at room temperature: for T cells with mouse anti-rat TCRαβ (1:250, clone R73, BD Bioscience, San Jose, CA, USA), and for monocytes and macrophages with mouse anti-rat CD68 (1:250, clone ED1; Serotec, Raleigh, NC, USA). Spinal cord sections were incubated for 1 h at room temperature with rabbit anti-rat ionized calcium binding adaptor molecule 1 (Iba-1, 1:2000; Wako Chemicals USA, Richmond, VA, USA) for microglia/macrophages and mouse anti-rat glial fibrillary acidic protein (GFAP; 1:2000, Chemicon, Temecula, CA, USA) for astrocytes in PBS containing 5% BSA, 0.05% Tween-20, and 0.05% Triton-X, or overnight at 4°C with mouse anti-rat TCRαβ (1:250) in PBS (with 5% BSA and 0.05% Tween-20). For double staining of M1 and M2 macrophages, sciatic nerves were stained for M1 macrophages with mouse anti-rat CD68 (1:250, clone ED1; Serotec) and rabbit anti-iNOS (1:1000; Abcam, Cambridge, UK). For M2 macrophages, the sections were stained with anti-Iba-1 (1:2000; Wako Chemicals USA) and mouse anti-mouse/rat Arginase-1 (1:100, clone 19; BD biosciences) and incubated at 4°C overnight. Then, the sections were rinsed four times in PBS and incubated for 1 h with the secondary antibodies, Alexa Fluor 488 conjugated donkey anti-mouse (1:250, Jackson Immuno Research Laboratories) or Cy3 conjugated donkey anti-rabbit (1:400, Jackson Immuno Research Laboratories) in the same buffer as the primary antibody. The sections were rinsed again four times in PBS, and prolong gold anti-fade reagent with 4', 6-diamidino-2-phenylindole (DAPI) (Life Technologies, Mulgrave, VIC, Australia) was then applied prior to slides being cover slipped.

### Immunofluorescent image analysis

Slides were visualized with an Olympus fluorescence microscope and images captured using an Olympus DP73 camera and DP Controller software (Olympus, Tokyo, Japan). For each animal, slides containing each of four sciatic nerve sections, four DRG sections, and six spinal cord sections (rostro-caudal) were stained for a given antibody. In the injured sciatic nerve sections, six images were captured (two at site of injury, two at proximal to the injury, and two at distal to the injury) per section (total 24 images per slide/animal). For DRGs, four images were taken from areas containing cell bodies in each section (four sections/slide). For spinal cord, images of the dorsal horn and ventral horn were taken from both ipsilateral and contralateral sides of six different L4-L6 coronal sections per slide. All images were captured at × 20 magnification. The cells were then either counted manually (TCR αβ) using the cell counter plug-in, or where their numbers were too numerous, were analyzed by densitometry (ED1, GFAP, and Iba-1) using ImageJ software (National Institutes of Health, Bethesda, MD, USA). Densitometry measurements were made by adjusting the threshold of the images to exclude nonspecific fluorescence and measuring the percentage of immune positive areas. Cell counts or densitometry measurements from each image analyzed were averaged for each region for each animal. These data were plotted as the average of three to four rats of cell count or percentage area of immunoreactivity. Six images from each slide were captured from sections stained for M1 and M2 macrophages along the sciatic nerve section. Images were taken where the maximum number of positively stained cells presented at × 10 magnification, and cells were counted manually using ImageJ.

### Cell isolation and flow cytometry

Flow cytometry was performed with tissues collected on days 10 and 30 post-CCI. The nerve-injured rats were deeply anesthetized with isoflurane, and popliteal and inguinal lymph nodes and spleen were collected in PBS. Each tissue was processed separately to achieve single cell suspensions by pressing the spleen and lymph nodes through a 40-μm cell strainer (BD Bioscience, Franklins Lakes, NJ, USA) in PBS. Cell suspensions were centrifuged for 4 to 5 min at 800 × *g* at 4°C before discarding the supernatant. Red blood cell lysis was performed for spleen samples by re-suspending cells in Red Blood Cell Lysis Buffer (eBioscience, San Diego, CA, USA) for 4 to 5 min with occasional shaking. Cell suspensions were first washed in PBS, with a second wash in Roswell Park Memorial Institute (RPMI) media (Invitrogen, Mulgrave, VIC, Australia). The cells were incubated at 37°C in RPMI/10% fetal bovine serum (Invitrogen) for 1 h to allow adherence and removal of monocytes. Following incubation, the cells were counted and re-suspended in flow cytometry staining buffer (eBioscience). Cell surface markers were stained for 30 min at 4°C, with the following combinations of antibodies: mouse anti-rat CD4-FITC (eBioscience) and mouse anti-rat CD25-APC (eBioscience) or suitable isotype controls. Then cells were washed three times with MACS buffer (Miltenyl Biotec, San Diego, CA, USA) before being fixed overnight at 4°C with fixation/permeabilisation solution (eBioscience). The samples were then washed twice with permeabilisation buffer (eBioscience) and stained with rat anti-mouse/rat Foxp3-PE or isotype control antibodies in permeabilisation buffer for 30 min at 4°C. Finally, samples were washed three times with permeabilisation buffer and re-suspended in MACS buffer (Miltenyl Biotec, San Diego, CA, USA). The cells were then acquired and analyzed on a BD FACS CantoII flow cytometer and BD FACS DIVA software (BD Biosciences). A minimum of 100,000 events was acquired for each sample.

### Serum preparation, protein purification/quantification, and cytokine assay

The rats were deeply anesthetized with isoflurane and 1 mL of blood was collected from the left ventricle of the exposed heart at day 10 post-CCI. Sodium pentobarbital was then injected i.p. and animals subsequently perfused with heparinised saline. Sciatic nerves, DRGs, and spinal cords were collected and flash-frozen in liquid nitrogen before being stored at −80°C until processing. Serum was separated from collected blood by allowing clotting at 37°C for 1 h, followed by centrifugation at 400 × *g* for 20 min. The supernatant was then collected, and aliquots were stored at −80°C.

Nervous tissue was mechanically dissociated on ice with protease inhibitor and TM buffer (Total protein extraction kit from Millipore, Billerica, Massachusetts, USA) according to the manufacturer’s instructions and homogenized with Precelly’s homogenizer in 2.8 mm ceramic bead tubes (Geneworks, Hindmarsh, SA, Australia) twice for 20 s each time. Homogenized tissue was rotated at 4°C for 20 min and centrifuged at 13,000 × *g* at 4°C for 20 min. Supernatants were collected, and proteins were quantified using the EZQ protein quantification kit (Molecular probes/Life technologies, Mulgrave, VIC, Australia) according to the manufacturer’s instructions.

Serum and protein samples of sciatic nerves, DRGs, and spinal cord were used for cytokine and chemokine analysis using the Bio-Plex Pro Rat Cytokine 23-plex Assay kit on a Bio-Rad Bio-plex machine (Bio-Rad Laboratories, Hercules, CA, USA). Serum samples, diluted at 1:4 dilution with sample diluent, and protein samples prepared to a concentration of 25 μg/50 μL, were analyzed per the manufacturer’s instructions.

### Experimental design and statistical analysis

**Figure 1:**
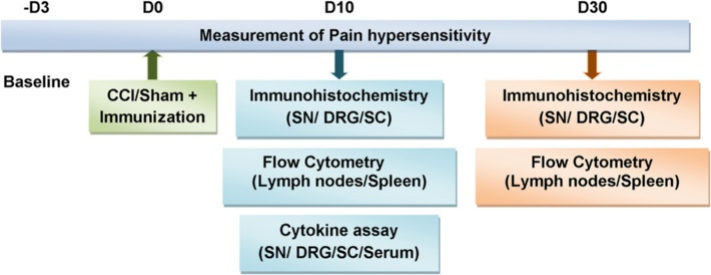
**A scheme of the experimental paradigm.** Male Lewis rats underwent CCI of the left sciatic nerve or sham operation on day 0 followed by subcutaneous immunization at the base of the tail. They were tested for pain hypersensitivity for 4 weeks (*n* = 6/group). On 10 and 30 days post-CCI, sciatic nerves (SNs), DRGs, and spinal cords (SCs) were collected for immunohistochemistry (*n* = 4/group) and spleens and lymph nodes for flow cytometry. For cytokine assay, SNs, DRGs, SCs, and serum were isolated from nerve-injured rats at 10 days post-CCI and from naïve rats (*n* = 6 to 7/group).

Details of the experimental design used to determine the effects of immunization on neuropathic pain and neuroinflammation are described in Figure 1. Following CCI/sham surgery, animals were randomly allocated to treatment groups and immunized and tested for pain hypersensitivity over 27 days. The number of animals used was *n* = 6 per group for pain measurements, *n* = 4 to 7 per group for cytokine assay, and *n* = 3 to 4 per group for immunohistochemistry and flow cytometry based on previous studies [6,12,13,28,33]. Tissue harvesting was carried out at days 10 and 30 post-CCI. Day 10 was chosen since pain hypersensitivity is well-established by day 10 after nerve injury and since we observed high withdrawal thresholds in the APL-treated rats with most significant differences between the groups on that day. At around 27 days after injury, the effects of immunization on pain responses started to decline, and therefore, day 30 was used as the end point of our experiment. The uninjured contralateral nerves and the contralateral side of the DRGs and spinal cords were used as internal controls in immunohistochemistry analysis, and serum and nervous tissues from naïve uninjured animals were used as controls in cytokine assays.

Statistical analysis was performed using Graphpad prism 6.0 software. Behavioral results were analyzed by two-way repeated measure analysis of variance (RM-ANOVA) followed by Tukey’s multiple comparisons post-test. Immunohistochemistry results and flow cytometry results were analyzed by two-way ANOVA with Tukey’s multiple comparison test. One-way ANOVA was used to analyze cytokine assay results with Dunnet’s multiple comparison test and multiplicity adjusted *P* values. *P* ≤ 0.05 was considered as statistically significant.

## Results

### Immunization with cyclo-(87-99)[A^91^_,_A^96^]MBP_87-99_ significantly decreased mechanical allodynia in the ipsilateral hindpaw of the nerve-injured rats

**Figure 2:**
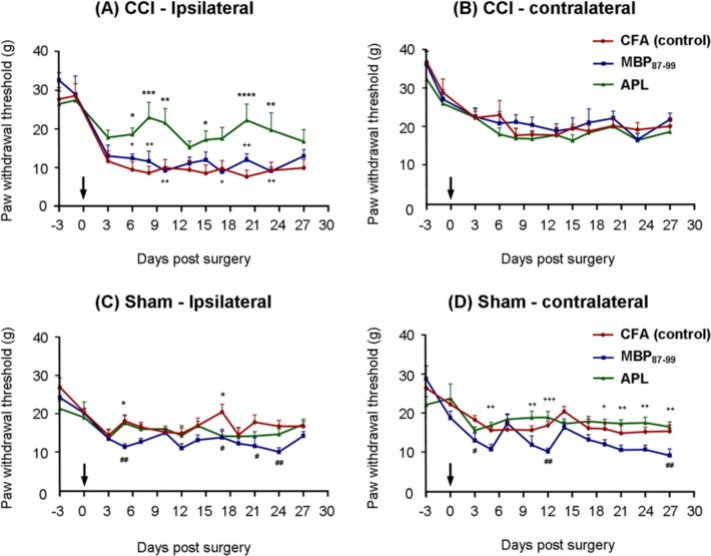
**Immunization with cyclo-(87-99)[A** ^**91**^ **,A** ^**96**^ **]MBP**_**87-99**_ **significantly decreases mechanical pain hypersensitivity following CCI. (A-D)** Time course of mechanical withdrawal threshold of the hindpaws (in grams) in nerve-injured and sham-operated rats treated with MBP_87-99_ (native peptide), APL cyclo-(87-99)[A^91^_,_A^96^]MBP_87-99_ and CFA only (vehicle control) in **(A)** the ipsilateral hindpaw and **(B)** the contralateral hindpaw of the rats with CCI and **(C)** ipsilateral hindpaw and **(D)** contralateral hindpaw of the sham-operated rats. +(*P* < 0.05), ++(*P* < 0.01), and ^+++^(*P* < 0.001) represent significant differences between the MBP- and APL-treated rats, *(*P* < 0.05), **(*P* < 0.01), ***(*P* < 0.001), and ****(*P* < 0.0001) indicate significant differences between the APL-treated and control rats. ^#^(*P* < 0.05) and ^##^(*P* < 0.01) represent significant differences between MBP-treated and control rats (*n* = 6 per group; mean ± SEM; two-way RM-ANOVA with Tukey’s multiple comparison test). Arrows indicate the time point of CCI/sham and immunization.

To investigate the effects of immunization with myelin-derived APL on pain hypersensitivity, we carried out CCI of the sciatic nerve followed by immunization with MBP_87-99_ (native peptide), APL cyclo-(87-99)[A^91^_,_A^96^]MBP_87-99_, and CFA only (vehicle control). Pain testing was carried out on both ipsilateral and contralateral hindpaws of the nerve-injured and sham-operated rats for 4 weeks post-surgery. The animals immunized with the MBP-derived APL demonstrated significantly decreased mechanical pain hypersensitivity in the left hindpaw compared to the MBP-treated and CFA-treated rats starting from day 6 until day 24 post-CCI (Figure 2A), with the most significant difference observed on days 8 (*P* < 0.001), 10 (*P* < 0.01), 20 (*P* < 0.0001), and 23 (*P* < 0.01) post-CCI. For example, on day 20, the APL-treated rats demonstrated a withdrawal threshold of 21.8 ± 4.1 g in the ipsilateral hindpaw, as compared to CFA-treated and MBP-treated rats, which had thresholds of 7.6 ± 0.6 and 11.8 ± 1.5 g, respectively. Nerve-injured rats started to develop pain hypersensitivity as early as 3 days after the injury. During the testing period, APL-treated rats’ paw withdrawal thresholds always remained in the range between 15 and 22.5 g, whereas with the MBP-treated and control rats, the maximum withdrawal threshold did not exceed 11.8 g at any time point. There were no differences between groups in mechanical pain sensitivity in the contralateral hindpaws (Figure 2B). We also examined the effect of immunization in the sham-operated rats. Interestingly, at several time points during the experiment, the sham-operated rats treated with MBP demonstrated elevated and statistically significant mechanical pain hypersensitivity compared to the APL-treated (*P* < 0.001) and CFA-treated (*P* < 0.01) sham rats in both ipsilateral (Figure 2C) and contralateral (Figure 2D) hindpaws. Nerve-injured rats did not develop significant thermal pain hypersensitivity, and therefore, no effects of immunization on thermal hyperalgesia were observed (data not shown). Further, animals immunized with either MBP or APL did not develop any sign of clinical EAE during the experimental period.

### Immunization with cyclo-(87-99)[A^91^_,_A^96^]MBP_87-99_ significantly reduced T cell infiltration to the injured sciatic nerve at 10 days post-CCI

**Figure 3:**
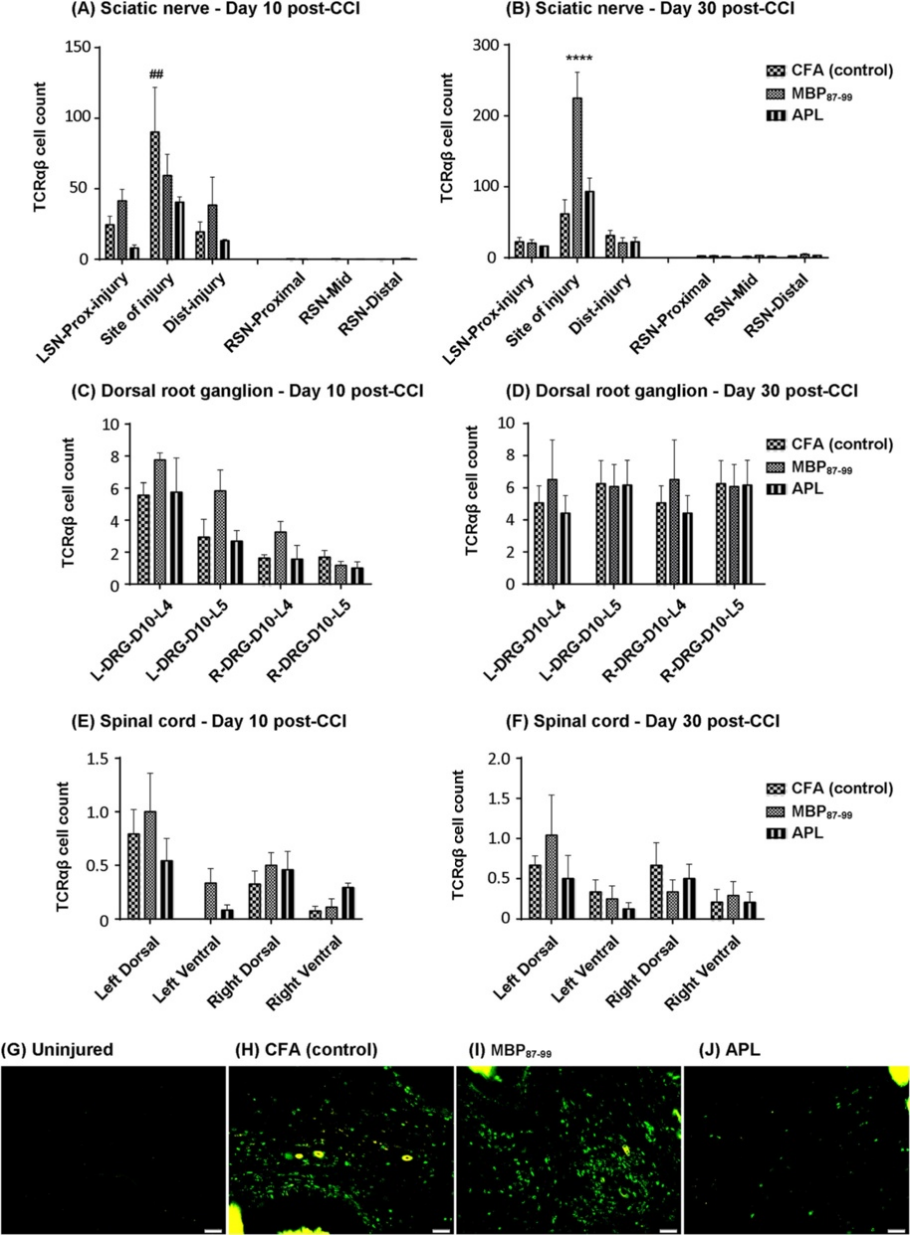
**Immunohistochemistry of T cells in the sciatic nerve, DRGs, and spinal cord.** The number of TCRαβ immunoreactive cells at the site of injury, proximal to the injury and distal to the injury in the left sciatic nerve (LSN) and uninjured right sciatic nerve (RSN) at 10 days **(A)** and 30 days **(B)** post-CCI. The number of TCRαβ immunoreactive cells in the L4 and L5 ipsilateral DRG (L-DRG) and contralateral DRG (R-DRG) at 10 days **(C)** and 30 days **(D)** post-CCI. The number of TCRαβ immunoreactive cells in the ipsilateral (left) and contralateral (right) lumbar spinal cord at 10 days **(E)** and 30 days **(F)** post-CCI. ^##^ *P* < 0.01 represents a significant difference between the APL-treated and control rats at the site of injury at 10 days post-CCI in the LSN. ****P < 0.0001 denotes significant differences between MBP-treated rats as compared to the APL-treated and control rats at 30 days post-CCI at the site of injury (*n* = 4 per group; mean ± SEM; two-way ANOVA with Tukey’s multiple comparison test). Representative images of **(G)** T cells in the uninjured nerve, **(H)** T cells in control, **(I)** T cells in MBP-treated and **(J)** T cells in APL-treated LSN at the site of injury. Scale bar = 50 μm.

T cells have been implicated in neuropathic pain [6] and MBP-derived APLs have been shown to modulate T cell responses in animal models [32]. Immunostaining for TCRαβ was performed on histological sections of the left and right sciatic nerves, L4 and L5 DRGs, as well as in the corresponding region of the lumbar spinal cord dissected at days 10 and 30 post-CCI and immunization. We found a significant reduction in T cell numbers in the injured sciatic nerve in rats treated with cyclo-(87-99)[A^91^_,_A^96^]MBP_87-99_, as compared to CFA-treated rats at 10 days post-CCI (Figure 3). T cell numbers were significantly increased in the CFA control group on 10 days post-CCI (Figure 3A) and in the MBP-treated group on 30 days post-CCI (Figure 3B) at the site of injury. On 10 days post-CCI, the T cell numbers at the site of injury were 90.2 ± 31.8 (CFA-treated; Figure 3H), 59.3 ± 15.0 (MBP-treated; Figure 3I), and 40.4 ± 3.8 (APL-treated; Figure 3J), representing a significant difference between CFA and APL groups (*P* < 0.01). Further, in the APL-treated group, the T cell counts proximal and distal to the injury were also lower than the other two groups at 10 days post-CCI, although the difference was not statistically significant (Figure 3A). On day 30 post-CCI (Figure 3B), the T cell numbers at the site of injury had sharply increased in the MBP-treated group (224.8 ± 36.6) compared to that on day 10 post-CCI (59.3 ± 15.0; *P* < 0.001). Further, APL-treated and control rats showed significantly lower (*P* < 0.0001) T cell count at the injured site (CFA = 61.9 ± 19.6 and APL = 93.2 ± 19) compared to MBP-treated group, but there was no significant difference in T cell count between the APL-treated and the control group at 30 days post-CCI. There was no significant difference between treatment groups at the proximal or distal to the injury site at day 30 post-CCI. Almost no T cells were found in the contralateral nerve at any time point in any of the treatment groups (Figure 3A-B,G). T cell infiltration to L4-L5 DRGs (Figure 3C-D) and dorsal and ventral horns of the spinal cord (Figure 3E-F) was comparatively very low compared to those of the injured sciatic nerve, and no significant differences were found between the groups.

### Immunization with cyclo-(87-99)[A^91^_,_A^96^]MBP_87-99_ significantly reduced ED1+ macrophage infiltration to the injured sciatic nerve

**Figure 4:**
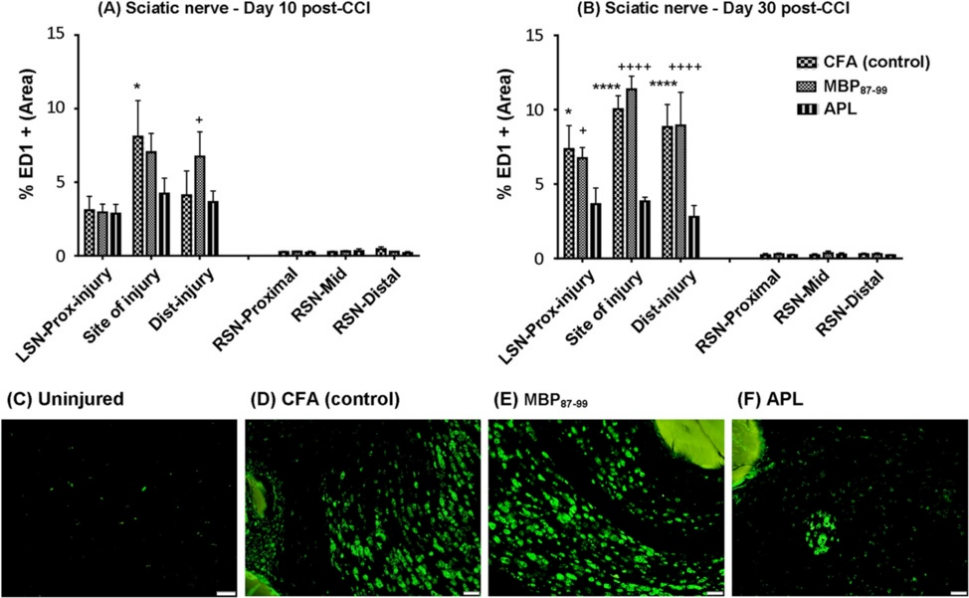
**Immunohistochemistry of ED1+ macrophages in the sciatic nerve.** The average of ED1+ area density at the site of injury, proximal to the injury and distal to the injury in the left sciatic nerve (LSN) and right uninjured nerve (RSN) at 10 days **(A)** and 30 days **(B)** post-CCI. *P < 0.05 represents significant difference between APL-treated and control rats at the site of injury and ^+^ *P* < 0.05 between MBP- and APL-treated rats distal to the injury at 10 days post-CCI. At 30 days post-CCI, *P < 0.05 represents significant difference between the APL and control group and ^+^ *P* < 0.05 between MBP and APL group proximal to the injury. ****P < 0.0001 represents significant differences between the APL and control group, ^++++^ *P* < 0.0001 between MBP and APL group at the site of injury and distal to the injury; (*n* = 4 per group; mean ± SEM; two-way ANOVA with Tukey’s multiple comparison test). Representative images of **(C)** ED1+ cells in uninjured RSN, **(D)** ED1+ cells in CFA-treated control, **(E)** ED1+ cells in MBP-treated, and **(F)** ED1+ cells in the APL-treated LSN at the site of injury. Scale bar = 50 μm.

Macrophages have also been shown to contribute to neuropathic pain behavior in animal models [34-36]. Thus, we stained injured and uninjured sciatic nerves with ED1, a generic macrophage marker, to investigate the effects of immunization on macrophage infiltration following nerve injury. We analyzed the percentage of ED1+ immunoreactivity at the site of injury, proximal to the injury and distal to the injury at 10 and 30 days post-CCI (Figure 4). Immunization with APL cyclo-(87-99)[A^91^_,_A^96^]MBP_87-99_ led to a significantly decreased percentage of ED1+ cells in the injured nerve (Figure 4A-B,F) in contrast to MBP-treated (Figure 4 A-B,E) and CFA-treated rats (Figure 4A-B,D). At 10 days post-CCI, immunization with the APL significantly decreased ED1+ macrophages compared to CFA-treated rats (P < 0.05) at the site of injury and compared to MBP-treated rats (P < 0.05) distal to the injury (Figure 4A). At 30 days post-CCI, APL immunization significantly reduced ED1+ cell reactivity proximal to the injury (P < 0.05), at the site of injury (P < 0.0001) and distal to the injury (P < 0.0001) compared to the control and MBP-treated rats (Figure 4B). Macrophages were found in the contralateral nerve in small numbers; however, there were no significant differences between treatment groups (Figure 4A-C).

### Immunization with cyclo-(87-99)[A^91^_,_A^96^]MBP_87-99_ significantly increased M2 macrophages in the injured nerve at day 30 post-CCI

**Figure 5:**
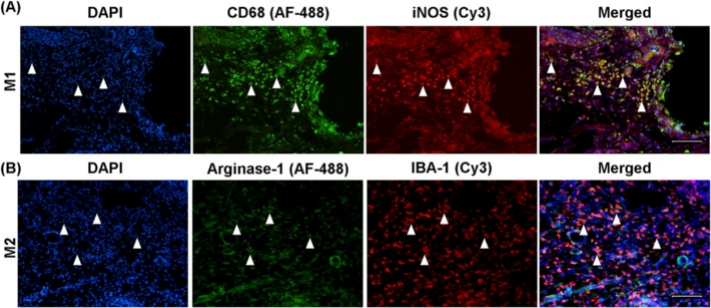
**Immunohistochemistry of M1- and M2-like macrophages in the injured nerve.** Representative immunofluoroscent images of triple labeled cells for **(A)** ED1+ (green) iNOS+ (red) DAPI (blue) and **(B)** Iba-1+ (red) Arginase-1+ (green) DAPI (blue) cells at the site of injury in the LSN. Scale bar = 100 μm.

Recently, several functional phenotypes of macrophages have been demonstrated, such as the M1/M2 paradigm comprised of pro-inflammatory (M1-polarized) and alternatively activated (M2-polarized) macrophages [37]. However, the activation of different subsets of macrophages following CCI has not been studied. Here, we double stained the injured sciatic nerves with ED1/iNOS (M1-like macrophages) and Iba-1/Arginase (M2-like macrophages) to investigate the effects of immunization with cyclo-(87-99)[A^91^_,_A^96^]MBP_87-99_ on M1/M2 macrophage expression following CCI. ED1+ and Iba-1+ cells were distributed all along the nerve either as clusters of cells or free standing cells; however, the distribution of double-stained cells was somewhat different. ED1+ iNOS+ cells (Figure 5A) were mainly located in the lesion itself or peri-lesion area as free-standing cells. In contrast, Iba-1+ Arginase + cells (Figure 5B) were found in the intact nerve, periphery, or peri lesion area either as cell clusters or single cells.

**Figure 6:**
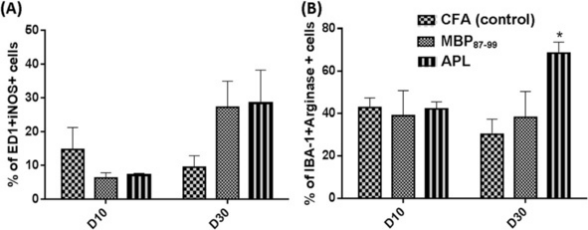
**Analysis of M1 and M2-like macrophages in the injured nerve.** The percentage of **(A)** ED1+ and iNOS+ cells (M1 macrophages) and **(B)** Iba-1+ and Arginase + cells (M2 macrophages) at 10 and 30 days post-CCI in the LSN. *P < 0.05 represents a significant difference between APL-treated and control rats (*n* = 3 per group; mean ± SEM; two-way ANOVA with Tukey’s multiple comparison test).

The percentages of M1-like macrophages were comparatively low at 10 days post-CCI in all treatment groups ranging from 10 to 15% of ED1+ cells, but were increased at 30 days post-CCI in both MBP-treated and APL-treated groups (to approximately 30%; Figure 6A). However, a statistically significant difference was not seen between the treatment groups. At 10 days post-CCI, the percentage of Iba-1+ Arginase + cells was around 40% in all groups (Figure 6B). Interestingly, at 30 days post-CCI, the APL-treated group had a significantly increased (*P* < 0.05) Iba-1+ Arginase + (M2) cell percentage compared to other treatment groups (Figure 6B).

### Effects of immunization on glia activation in the lumbar spinal cord

**Figure 7:**
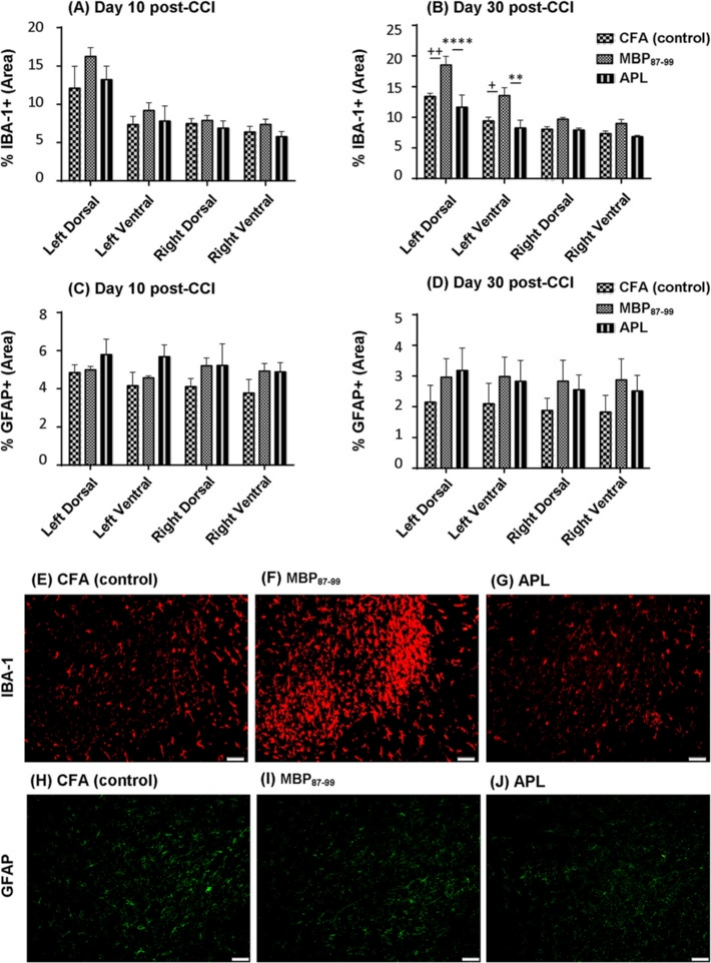
**Immunohistochemistry of microglia and astrocytes in the lumbar spinal cord following CCI and immunization.** The percentage of Iba-1+ area density in the dorsal and ventral lumbar spinal cord at 10 days **(A)** and at 30 days **(B)** post-CCI and the percentage of GFAP+ area density in the dorsal and ventral lumbar spinal cord at 10 days **(C)** and at 30 days **(D)** post-CCI. Representative images of Iba-1+ cells in the spinal cord of the control **(E)**, MBP-treated **(F)**, APL-treated **(G),** and GFAP+ cells in the spinal cord of the control **(H)**, MBP-treated **(I)**, and APL-treated **(J)** rats. ^+^ *P* < 0.05 and ^++^ *P* < 0.01 represent significant differences between the MBP-treated and control rats, and **P < 0.01 and ****P < 0.0001 indicate significant differences between MBP- and APL-treated rats at 30 days post-CCI (*n* = 4 per group; mean ± SEM; two-way ANOVA with Tukey’s multiple comparison test).

It has been shown that in models of peripheral nerve injury, the glial cells in the spinal cord become activated and contribute to the development of neuropathic pain [13]. Spinal cord sections were stained with Iba-1 to detect activation of resident microglia and infiltrated macrophages and with GFAP to detect activated astrocytes in the spinal cord following CCI and immunization (Figure 7). Data revealed a significant increase in Iba-1 immunoreactivity in the MBP-treated group compared to the other groups on day 30 (Figure 7B) but not on day 10 (Figure 7A) post-CCI in the ipsilateral dorsal and ventral horn of the spinal cord. In the left dorsal horn, the MBP-treated rats (Figure 7B,F) exhibited significantly increased microglia activation compared to the CFA group (Figure 7 B,E; *P* < 0.01) and APL group (Figure 7B,G; *P* < 0.0001). Similarly, significant differences were seen in the left ventral horn between the MBP-treated group and CFA-treated group (*P* < 0.05), as well as the MBP-treated and APL-treated groups (*P* < 0.01) on 30 days post-CCI. However, no significant differences in Iba-1 immunoreactivity were found between the APL-treated (Figure 7A-B,G) and control groups (Figure 7A-B,E).

GFAP immunoreactivity in the ipsilateral and contralateral sides of the spinal cord was higher on day 10 post-CCI compared to day 30 post-CCI within the three treatment groups, but there were no significant differences between groups on each of the tested days (Figure 7C-D,H-J).

### Immunization with cyclo-(87-99)[A^91^,A^96^]MBP_87-99_ increased splenic regulatory T cells in the nerve-injured rats at 10 days post-CCI

**Figure 8:**
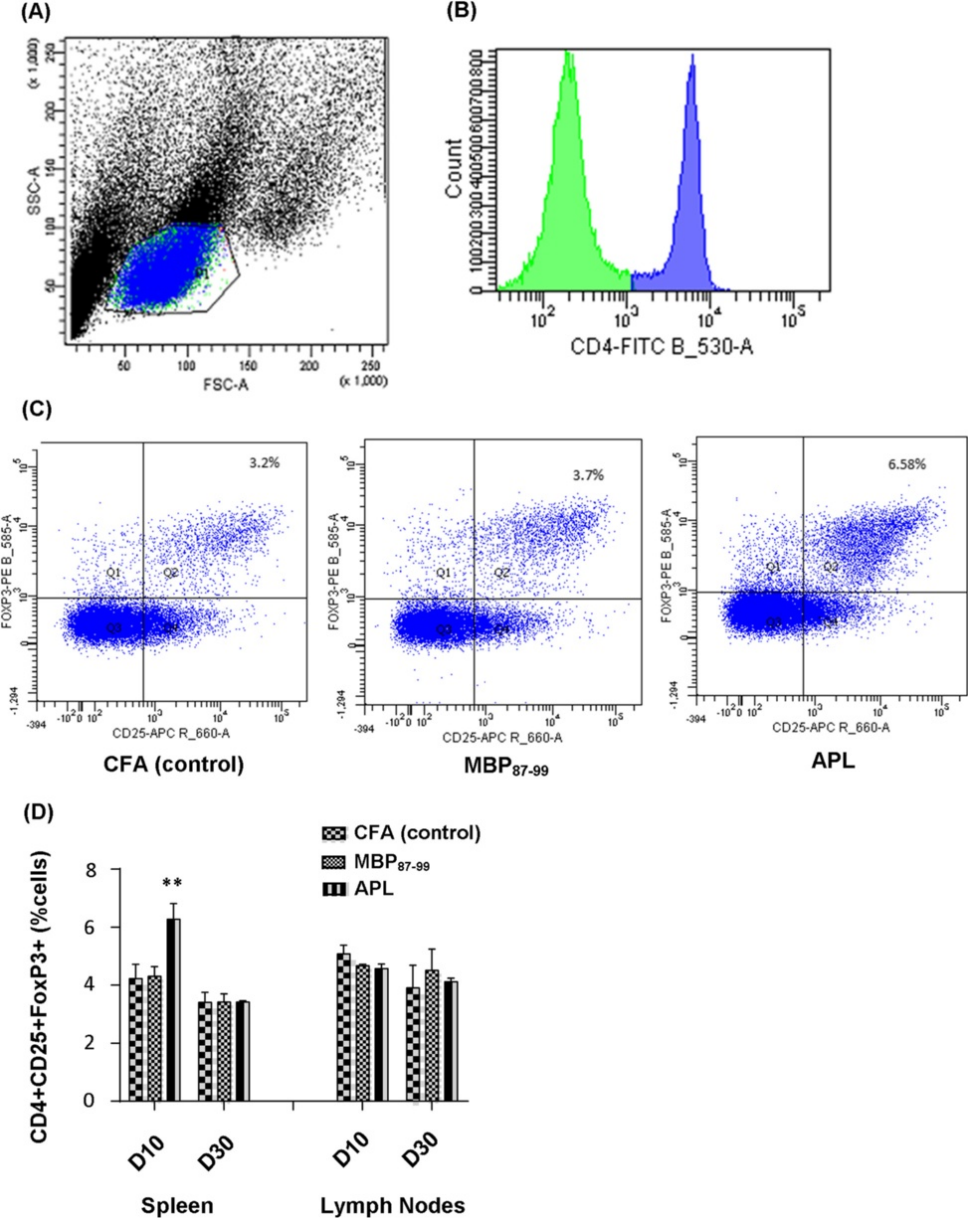
**Flow cytometry analysis of CD4+ CD25+ FoxP3+ expressing Treg cells in the spleen and lymph nodes following CCI and immunization. (A)** Representative forward scatter (FSC) vs. side scatter (SSC) plot showing the acquired events and lymphocyte gate from rat spleen. **(B)** Representative histogram of counts vs. CD4+ cell population selected for further gating. **(C)** Representative dot plots of Treg cells in the spleen of the control (left), MBP-treated (middle), and APL-treated (right) rats at 10 days post-CCI. **(D)** The percentage of CD4+ CD25+ FoxP3+ cells in the spleen and lymph nodes at 10 and 30 days post-CCI. **P < 0.01 indicates significant differences between APL-treated and MBP-treated and control rats at 10 days post-CCI (*n* = 4 per group; mean ± SEM; two-way ANOVA with Tukey’s multiple comparison test).

Previous reports showed that treatment with antagonistic APLs can induce immunosuppressive regulatory T cells [20]. To investigate the effect of immunization with cyclo-(87-99)[A^91^_,_A^96^]MBP_87-99_ on the prevalence of Treg cells in the spleen and lymph nodes, we used flow cytometric analysis at day 10 and 30 post-CCI (Figure 8). To identify regulatory T cells, we first gated CD4+ cells (Figure 8B) out of lymphocyte singlets (Figure 8A) in a total population of cells and then further gated for CD25+ and FoxP3+ cells (Figure 8C). We found that the percentage of Treg cells in the spleen at 10 days post-CCI was significantly increased (*P* < 0.01) in the APL-treated group compared to the MBP-treated and control rats (Figure 8C-D). No significant differences between treatment groups were seen in the spleen at 30 days post-CCI (Figure 8D, left) or in the lymph nodes at any time point analyzed (Figure 8D, right).

### Effects of immunization with MBP_87-99_ and cyclo-(87-99)[A^91^_,_A^96^]MBP_87-99_ on cytokine expression following CCI

**Figure 9:**
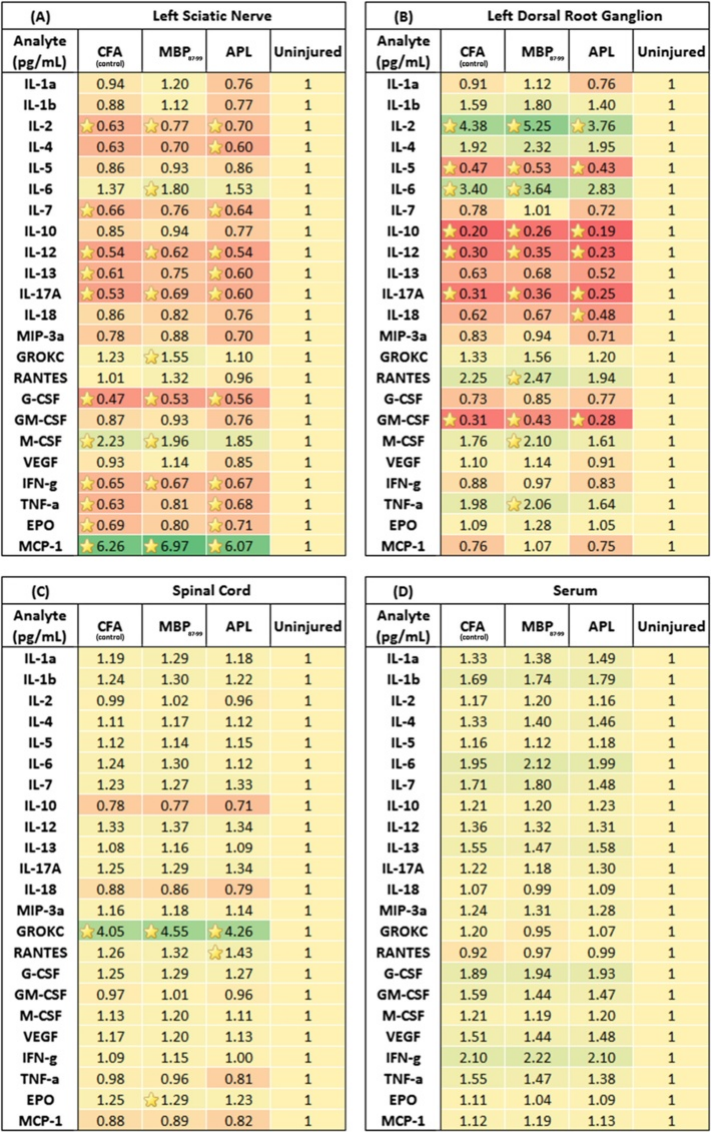
**Bio-plex analysis of cytokine expression profile in the nervous tissue and serum at 10 days post-CCI.** Heat maps illustrating the summary of fold change expression of cytokines in the **(A)** LSN, **(B)** left DRG, **(C)** spinal cord, and **(D)** serum of immunized nerve-injured rats compared to control uninjured naïve rats at 10 days post-CCI. Yellow asterisks indicate significant differences in cytokine concentrations in treatment groups as compared to the uninjured control group (*n* ≥ 4 per group; one-way ANOVA with Dunnet’s multiple comparison test compared to the uninjured group).

**Figure 10:**
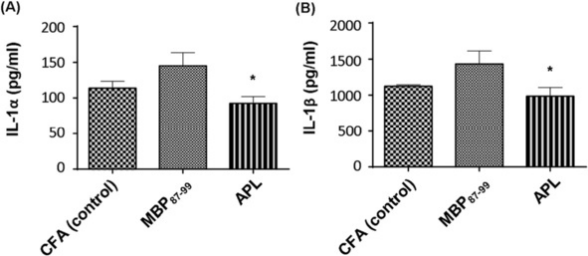
**IL-1α and IL-β profile in the injured sciatic nerve at 10 days post-CCI.** The concentrations of IL-1α **(A)** and IL-1β **(B)** in the LSN of immunized nerve-injured rats at 10 days post-CCI. *P ≤ 0.05 indicates a significant difference between MBP- and APL-treated rats (*n* = 6 per group; mean ± SEM; one-way ANOVA with Dunnet’s multiple comparison test compared to the APL-treated group).

To test the effect of immunization with MBP_87-99_ and cyclo-(87-99)[A^91^_,_A^96^]MBP_87-99_ on cytokine expression in the serum and relevant nervous system tissues, we used a multiplex cytokine assay. In the injured left sciatic nerve, the cytokines IL-2, IL-4, IL-7, TNF-α, IFN-γ, granulocyte colony stimulating factor (G-CSF), erythropoietin (EPO), IL-17A, IL-13, and IL-12 were significantly (*P* < 0.05 to 0.001) reduced, whereas the cytokines IL-6, keratinocyte chemo attractant/growth-related oncogene (GRO/KC), macrophage colony stimulating factor (M-CSF), and monocyte chemoattractant protein-1 (MCP-1) were significantly (*P* < 0.05 to 0.0001) increased in at least one treatment group relative to the uninjured nerve. No significant differences were found in IL-1α, IL-1β, IL-5, IL-10, IL-18, macrophage inflammatory protein (MIP)-3a, regulated on activation normal T cell expressed and secreted (RANTES), granulocyte macrophage colony stimulating factor (GM-CSF), and vascular endothelial growth factor (VEGF) in the injured nerve of all treatments groups compared to the uninjured nerve (Figure 9A). In the ipsilateral DRG, the cytokines IL-5, IL-10, IL-12, GM-CSF, and IL-17A were significantly reduced (*P* < 0.05 to 0.001), whereas IL-2, IL-6, M-CSF, RANTES, and TNF-α (*P* < 0.001) were significantly increased in at least one of the treatment groups relative to the uninjured DRG. The cytokines IL-1, IL-4, IL-7, IL-13, IL-18, MIP-3a, GRO/KC, G-CSF, M-CSF, VEGF, IFN-γ, EPO, and MCP-1 in the ipsilateral DRG in all treatment groups were not significantly changed compared to the uninjured DRG (Figure 9B). In the spinal cord, concentrations of GRO/KC (*P* < 0.0001), RANTES, and EPO (*P* < 0.05 to 0.01) were significantly increased in at least one of the treatment groups relative to the uninjured spinal cord. All other cytokines were not significantly changed in the spinal cord of the treated rats as compared to the spinal cord of the uninjured rats (Figure 9C). In the serum, there was no significant difference in any of the cytokines tested in all treatment groups relative to the serum of normal rats (Figure 9D). Interestingly, the concentrations of IL-1α (Figure 10A) and IL-β (Figure 10B) in the injured nerve of MBP-treated rats significantly increased (IL-1α *P* = 0.02 and IL-1β *P* = 0.04; one-way ANOVA followed by Dunnet’s multiple comparison test with adjusted *P* value) compared to APL-treated rats, but not compared to the control CFA-treated rats. There were no statistically significant differences between APL-treated and control rats in any of the cytokines tested.

## Discussion

The protective effects of antagonistic myelin-derived APLs through T cell modulation have been demonstrated in several models of autoimmune disease and spinal cord injury [18,24,38,39], suggesting a potential use in animal models of neuropathic pain. Here, we investigated the effects of active immunization with cyclic MBP_87-99_ (native peptide) and cyclo-(87-99)[A^91^,A^96^]MBP_87-99_ (APL) on pain sensitivity and neuroinflammation following peripheral nerve injury in rats. We demonstrate that immunization with cyclo-(87-99)[A^91^,A^96^]MBP_87-99_ reduces mechanical pain hypersensitivity in the ipsilateral hindpaws in association with reduced T cell infiltration at the site of injury, increased presence of M2 macrophages in the injured nerve, unaltered microglial activation in the ipsilateral lumbar spinal cord, and increased prevalence of splenic Treg cells. In agreement with these findings, MBP-derived APLs have been shown to modulate inflammatory responses towards an anti-inflammatory mechanism [32] as well as to increase Treg cells in previous studies [21]. Our result of reduced mechanical allodynia in rats immunized with cyclo-(87-99)[A^91^,A^96^]MBP_87-99_ is consistent with our previous report that immunization with the same APL significantly reduced mechanical allodynia in Lewis rats with EAE [24]. In addition, immunization with the native peptide of MBP induced some mechanical allodynia in the sham-operated rats. A recent study by Liu et al. indicates MBP as a novel mediator of pain, as administration of MBP_69-86_ or MBP_84-104_ peptides into naïve nerve produced robust mechanical allodynia with an associated increase in immune cell trafficking and antigen presentation in the injected nerve. These effects were considerably reduced in T cell-deficient nude rats, indicating the involvement of T cells in MBP-mediated pain [28]. Although we used a different epitope of MBP, it may have contributed to the mechanical pain hypersensitivity exhibited by the sham-operated rats in our study. A previous work demonstrated that cyclo-MBP_87-99_ induces, while the antagonistic APLs cyclo-(91-96)[Ala^96^]MBP_87-99_ and cyclo-(87-99)[Arg^91^,Ala^96^]MBP_87-99_, inhibit T cell proliferation in an *in vitro*-generated CD4+ T cell line [23]. This suggests that immunization with MBP may have caused recruitment of MBP-specific T cells to the injured nerve. Conversely, such recruitment might have been suppressed in APL-treated rats. Indeed, following peripheral nerve injury matrix metalloproteinases promote peripheral myelin damage through degradation of endogenous MBP, as well as macrophage infiltration and central glia activation, thus contributing to mechanical allodynia [27]. The repeated exposure of MBP epitopes caused by the injury-induced MBP degradation leads to the formation of MBP-specific T cell clones and a self-sustaining immune reaction [28], which may be further facilitated by immunization with MBP and inhibited by immunization with APL.

T cell infiltration into the injured nerve has been associated with the development of pain hypersensitivity, and animals lacking functional T cells have reduced pain responses following peripheral nerve injury [5,6]. In accordance with this, we found that T cell infiltration to the injured nerve was lower in rats immunized with cyclo-(87-99)[A^91^,A^96^]MBP_87-99_ at 10 days post-CCI, which also showed reduced mechanical pain hypersensitivity. APLs can act as partial T cell agonists and thereby result in either T cell anergy (functional incapacity of T cell response to antigen) [40], down-regulation of effector T cells [41,42], or mediate bystander suppression through the generation of Treg cells [20]. Thus, the above mechanisms may be involved in the decreased T cell infiltration into the injured nerve, and the lessened mechanical allodynia in the APL-treated nerve-injured rats. Although the dynamics of T cell infiltration into the injured nerve differed among the treatment groups, T cell numbers in the injured nerve were considerably higher relative to the uninjured nerve in all the groups. However, we found a significant increase in T cell counts in the control rats at 10 days post-CCI and in the MBP-treated rats at 30 days post-CCI when compared to APL-treated rats. Indeed, a previous study demonstrated that T cells infiltrate the injured nerve after CCI in increasing numbers up to 21 days post-CCI followed by a gradual reduction towards 40 days post-CCI in Lewis rats [6]. Since MBP is expressed in both the peripheral and central nervous system, nerve injury may have exposed cryptic MBP epitopes [28] resulting in continuous activation of MBP-specific T cells over time. This may explain the pronounced increase in T cell infiltration in MBP-treated rats at 30 days post-CCI. Although the activation of T cells in the dorsal spinal cord following peripheral nerve injury has been implicated in neuropathic pain [5,43], significant numbers of T cells and differences between treatment groups in the spinal cord and DRGs were not seen in this study. Previous studies have demonstrated that immunization with APLs increase Treg cells and mediate bystander suppression in animal models of EAE [20]. We previously showed that Treg cell expansion reduced mechanical pain hypersensitivity following CCI [13]. Our current study shows a significant elevation of Treg cells in the spleen of APL-treated nerve-injured rats at 10 days post-CCI. Although the antigen specificity and functional activity of these Treg cells is unknown, increased splenic Treg cells in the cyclo-(87-99)[A^91^,A^96^]MBP_87-99_-treated group may have conferred a beneficial effect on neuropathic pain behavior. The mechanism by which this is accomplished is unclear but may be due to suppression of conventional antigen-specific T cells and killing T cells by granzyme-dependent or perforin-dependent mechanisms [44] in the periphery.

ED1+ macrophage reactivity in the injured nerve was significantly reduced in cyclo-(87-99)[A^91^,A^96^]MBP_87-99_-treated animals. Indeed, macrophage depletion has been shown to attenuate pain hypersensitivity in some neuropathic pain models [34,36]. Recently however, the concept of formation of definitive functional macrophage phenotypes has emerged. M1 activity is associated with inhibition of cell proliferation and tissue damage (predominantly pro-inflammatory), while M2 activity is linked to the promotion of cell proliferation and tissue repair (predominantly anti-inflammatory) [37,45,46]. Therefore, we further investigated the effect of treatment on the prevalence of M1 and M2 macrophages in the injured nerve. ED1+ cells and Iba-1+ cells were visible along the length of the nerve; however, iNOS+ macrophages were mostly localized at the site of injury, and Arginase+ cells were localized in the intact nervous tissue and towards the periphery of the nerve. This kind of cellular pattern clearly suggests the pro-inflammatory nature of M1 macrophages being responsible for clearing debris at the site of injury and the anti-inflammatory role of M2 macrophages in protecting the surrounding tissue [46]. Interestingly, we observed a mixed infiltration of M1 and M2 macrophages in the injured nerve at both 10 and 30 days post-CCI regardless of treatment. M1 macrophages seemed to be less prevalent at 10 days post-CCI and increased over time, albeit there was no significant difference between the treatment groups. A previous study has investigated the expression of M1 and M2 markers following axotomy in the sciatic nerve in C57BL/6 mice [45]. They did not find any of the M1 markers including iNOS in the nerve at any time point investigated, which is contrary to our results. However, Turtzo et al. demonstrated characteristic expression of M1 and M2 markers in the brain following traumatic brain injury (TBI) in female Wistar rats [46]. Two other studies of TBI in mice have reported a mixed M1 and M2 response, in which M2 response peaked at 5 days post-CCI and returned to baseline by 2 weeks [47,48]. Similarly, a study of mice with spinal cord injury has shown expression of distinct M1 and M2 markers with M1 phenotype dominating the lesion site and nearby spared tissue and the M2 response lasting only for 3 to 7 days post-injury [37], whereas in our study, the Arginase + M2 response continued until 30 days post-CCI. These studies illustrate the diversity in macrophage activation across species and experimental models.

A previous study has demonstrated that immunization of spinal cord-injured mice with myelin oligodendrocyte glycoprotein (MOG)-derived altered peptide resulted in a higher number of recruited monocyte-derived macrophages with anti-inflammatory properties and improved recovery [49]. Herein, we show that treatment with cyclo-(87-99)[A^91^,A^96^]MBP_87-99_ has significantly increased the presence of M2 macrophages at 30 days post-CCI, suggesting a possible immunomodulatory mechanism of APL towards an M2-mediated phase in the latter stages following peripheral nerve injury. M2 macrophages are capable of resolving inflammation and promoting Th2 immune responses [50]. Previous studies reported inhibition of EAE, suppression of Th17 cell proliferation, upregulation of Th2 and Treg cells, reduced macrophage/microglia activation, and increased Treg cell gene expression such as GATA3 and FoxP3 in mice following adoptive transfer of M2 macrophages [51,52]. Such protective mechanisms of M2 macrophages may have contributed to the reduced neuroinflammation and mechanical allodynia in the APL-treated rats.

Microglial activation in the spinal cord plays a key role in driving the development and maintenance of pain hypersensitivity following peripheral nerve damage, and pharmacological inhibition of microglial activation has been shown to attenuate neuropathic pain [53,54]. In this study, the MBP-treated rats demonstrated significantly increased microglial activation in the ipsilateral lumbar spinal cord compared to the cyclo-(87-99)[A^91^,A^96^]MBP_87-99_-treated and control rats. However, despite a significant difference in pain hypersensitivity between APL-treated and control rats, there was no significant difference in their spinal microglial activation suggesting that microglia do not play a key role in ameliorating mechanical pain hypersensitivity in APL-treated nerve-injured rats. Other studies have also reported no correlation between neuropathic pain behaviors and microglial activation. For example, Colburn et al. demonstrated that neuropathic pain behaviors did not correlate with spinal microglial responses following nerve injury; in some rats, pain hypersensitivity existed in the apparent absence of microglial activation, and conversely, profound microglial activation was occasionally associated with a lack of pain hypersensitivity [55]. Likewise, perineural application of the local anesthetic bupivacaine markedly reduced microglia responses following spinal nerve cryoneurolysis without ameliorating pain hypersensitivity [55]. Other models of neuropathic pain, such as chemotherapy-induced peripheral neuropathy [56] and central pain due to oligodendrocyte ablation [57] have shown no correlation between microglial activation and pain responses. Similarly, several studies have demonstrated the presence of pain hypersensitivity in the absence of astrocyte activation [57,58], in line with the lack of spinal astrocyte activation in the present study. For example, a previous study has shown no change in GFAP-positive cells and GFAP protein levels in the ipsilateral dorsal horn of the spinal cord following CCI [59]. Robust mechanical pain hypersensitivity was elicited in oligodendrocyte-ablated mice without significant activation of astrocytes [57]. A preconditioning nerve lesion inhibited mechanical pain hypersensitivity following subsequent neuropathic injury without altering either spinal microglial or astrocyte activation [60]. It is plausible that our treatments induced a change in as yet unknown distinct phenotypes of glial cells without altering the total expression of activation markers in the spinal cord.

Treatment with APLs has also been shown to modulate the cytokine response in mice with EAE [32]. For example, Katasara et al. have previously demonstrated increased concentrations of anti-inflammatory cytokines IL-4 and IL-10 in SJL/J mice following intradermal immunization with MBP-derived APL conjugated to reduced mannan [32]. We found an altered expression of several cytokines in the injured nerve, ipsilateral DRG, spinal cord, and serum in all treatment groups as compared to control naïve animals. It is noteworthy that the profile of some cytokines was completely different in each of the different sites along the pain pathway. A surprising finding was a reduction in the expression of several pro-inflammatory cytokines in the nervous system tissues of nerve-injured animals. Turtzo et al. has also reported a similar pattern of cytokine expression with increased cytokine levels in the contralateral side in Wistar rats following TBI [46]. In fact, unilateral peripheral nerve injury has been shown to affect the contralateral side [61,62], and upregulated cytokine and chemokine gene expression has been observed in the contralateral nerve following CCI in mice [63]. The later study identified the involvement of N-methyl-D-aspartate (NMDA) receptor signaling as one underlying mechanism, since intraperitoneal administration of NMDA antagonist MK-801 completely blocked the contralateral gene expression of IL-1β and MCP-1 following CCI [63]. It is logical to assume that differences in cytokine expression between studies may result from differences in species, strain, injury type, testing time, tissue analyzed, and sampling and processing methods [64].

In the current study, we observed that MBP-treated rats had significantly increased IL-1α and IL-1β concentrations in the injured nerve analyzed at 10 days post-CCI compared to cyclo-(87-99)[A^91^,A^96^]MBP_87-99_-treated rats. The IL-1 family consists of IL-1α and IL-1β, which bind to the IL-1-type 1 receptor and the IL-1 receptor accessory protein [65]. IL-1β is one of the predominant pro-inflammatory cytokines released following injury [66] and is known to be released by microglia and macrophages [67]. The involvement of IL-1 in neuropathic pain has been previously shown [68,69], including its ability to increase neuronal excitability [70]. It has been reported that the intrathecal administration of IL-1β induces mechanical and thermal hyperalgesia in rats [71,72], whereas intrathecal administration of IL-1 receptor antagonists (IL-1ra) alleviated hyperalgesia in CFA-induced inflammatory pain [73]. In addition, deletion of IL-1 receptors and overexpression of IL-1ra ameliorated neuropathic pain behavior in transgenic mice with spinal nerve transection [74]. Thus, upregulation of IL-1β by MBP_87-99_ may have played a role in the mechanical pain hypersensitivity in MBP-treated rats in the present study.

## Conclusions

The present data suggest that immune deviation by active immunization with a non-encephalitogenic myelin-derived APL mediates an analgesic effect in neuropathic animals. The exact analgesic mechanisms of the APL cyclo-(87-99)[A^91^,A^96^]MBP_87-99_ in nerve-injured animals are not known, however, modulating neuroinflammation by decreased infiltration of T cells and macrophages, upregulation of a M2 macrophage-dominant anti-inflammatory response and upregulation of splenic regulatory T cells are all possible explanations. These findings support the potential application of immunization with myelin-derived APL for the treatment of peripheral neuropathic pain.

## Abbreviations

CCI: chronic constriction injury
CNS: central nervous system
CFA: complete Freund’s adjuvant
MBP: myelin basic protein
APL: altered peptide ligand
IL: interleukin
TNFα: tumor necrosis factor
PSNL: partial sciatic nerve ligation
TCR: T cell receptor
MS: multiple, sclerosis
EAE: experimental autoimmune encephalitis
MHC: major histocompatibility complex
IFN-γ: interferon-γ
LSN: left sciatic nerve
LDRG: left dorsal root ganglia
RSN: right sciatic nerve
RPMI: Roswell Park Memorial Institute
RM-ANOVA: repeated measures analysis of variance
GRO/KC: keratinocyte chemo attractant/growth-related oncogene
TBI: traumatic brain injury
PLP: proteolipid protein
MOG: myelin oligodendrocyte glycoprotein
PBS: phosphate-buffered saline
DAPI: 4',6-diamidino-2-phenylindole
GFAP: glial fibrillary acidic protein
IBA-1: ionized calcium-binding adapter molecule 1
IL-1ra: IL-1 receptor antagonist
G-CSF: granulocyte colony stimulating factor
EPO: erythropoietin
M-CSF: macrophage colony stimulating factor
MCP-1: monocyte chemoattractant protein-1
MIP-3a: macrophage inflammatory protein
RANTES: regulated on activation normal T cell expressed and secreted
GM-CSF: granulocyte macrophage colony stimulating factor
VEGF: vascular endothelial growth factor
PFA: paraformaldehyde
